# Amalgam and Matrix

**Published:** 1887-07

**Authors:** Frank W. Low

**Affiliations:** Buffalo, N. Y.


ARTICLE VIII.

### AMALGAM AND MATRIX.

BY DR. FRANK W. LOW, BUFFALO, N. Y.

Whatever may be urged for or against the use of the matrix in general, in operation for compound or crown approximal cavities, whether in bicuspids or molars, if amalgam is to be used as the filling material, the matrix ought to be considered an indispensable adjunct, because in no other way can the contour of the tooth be so perfectly restored, while the introduction of the filling is converted from a complicate to a very simple operation. The amalgam can be impacted and hardened by direct pressure, and so thoroughly burnished as would otherwise cause protrusion of the mass at the cervical walls; nor will there be any danger from mastication, even if put to use immediately upon completion of the operation.

By many who work amalgams of inferior quality, this last statement will be read with incredulity, and the objection will be raised that the removal of the matrix is liable to dislodge the filling. Let me detail a process which, if accurately followed, will both banish all doubt and overcome all objection:

The decay having been excavated and the cavity otherwise properly shaped, it is advisable, as a final precaution against the possibility of dislodgment, to make with a sharp fissure bur two marked dovetailed enlargements in the crown opening well back in the body of the tooth, one approaching toward its buccal, the other toward its lingual wall. When sufficient separation can readily be obtained, unless one may prefer to use the device invented by Dr. Wm. B. Miller, the Jack's matrix should perhaps be chosen in preference to all others, and as two sets—one thick, one thin—are now devised, they will meet the exigencies of a majority of cases. However, if the teeth approximate so closely that sufficient separation seems impossible, or if the separation is too great and the matrix cannot be held in position by impingement against the adjoining tooth, then the choice of a suitable band matrix is indicated. The Ladmore-Brunton clamps and matrices (an English device which has the advantage over its American competitors to public favor, in that it is adjusted and tightened with a flexible key) and those devised by Dr. S. H. Guilford, are much alike, while the Loop matrices, invented by Dr. Frank

Creager and by Dr. W. Pinney, more nearly resemble each other in the manner of their construction; and yet another, differing considerably from both the bands of Drs. Creager and Pinney, is the device of Dr. T. W. Brophy. These are each and all, in their several ways, excellent devices. He who has any one of them is pretty well equipped; he who has them all is truly fortified against any contingency.

To guard against the possibility of slipping, to insure perfect adaptation to the cervical walls, and to induce a slight additional separation for the easy removal of the matrix when the operation is completed, it is well to force a wedge of orange-wood (dipped in copal-ether varnish and so sharpened as to conform to the V-shaped space between the necks of the teeth) in between the matrix and the adjoining teeth. A magnifying glass should be used to inspect the cavity as soon as the wedge is adjusted and excised to make sure of this perfect adaptation, for it is an all-important requirement to the finished work.

In mixing the amalgam, great care should be taken to combine the alloy with *pure* mercury in definite proportions because the hardness of the plug, as well as the immunity from shrinkage, depends upon the perfect satisfaction of certain natural combining weights of the different metals with the mercury of amalgamation, so that either a lack of or surplus of mercury is disastrous to this perfect combination. The best method for mixing is that recommended by Dr. Thos. Fletcher, namely: "Put the amalgam in a glass mixing tube; cover the open end of the tube with the finger and shake briskly for a few seconds; work the resulting mass into little disks with a cylinder mould."

The writer invariably makes use of the little balance devised by Dr. Fletcher for combining amalgam in proper proportions; and prefers to all others his Platinum and Gold Alloy. The amalgams of other manufacturers may be equally good, but their proportions for perfect amalgamation should be determined and stated, so that they may be accurately weighed for combination as above recommended. No amalgam can be successfully used with a matrix that will not burnish hard as soon as the cavity is filled, and *no amalgam will allow of sufficicntly hard burnishing* if moisture is allowed to come in contact with portions of it while being introduced. On this account it is well, even after the operation is completed, before removing the rubber dam or napkin, to varnish the finished plug with copal-ether varnish.

A most bright and beautiful luster may be given amalgam (which in most instances remains for years) by reburnishing and polishing at a subsequent sitting, and no time is better spent, even by the busiest operator, than to beautify his work after completion.

